# Survival rates in a small hibernator, the edible dormouse: a comparison across Europe

**DOI:** 10.1111/j.1600-0587.2010.06691.x

**Published:** 2011-08

**Authors:** Karin Lebl, Claudia Bieber, Peter Adamík, Joanna Fietz, Pat Morris, Andrea Pilastro, Thomas Ruf

## Abstract

Understanding how local environmental factors lead to temporal variability of vital rates and to plasticity of life history tactics is one of the central questions in population ecology. We used long-term capture-recapture data from five populations of a small hibernating rodent, the edible dormouse *Glis glis*, collected over a large geographical range across Europe, to determine and analyze both seasonal patterns of local survival and their relation to reproductive activity. In all populations studied, survival was lowest in early summer, higher in late summer and highest during hibernation in winter. In reproductive years survival was always lower than in non-reproductive years, and females had higher survival rates than males. Very high survival rates during winter indicate that edible dormice rarely die from starvation due to insufficient energy reserves during the hibernation period. Increased mortality in early summer was most likely caused by high predation risk and unmet energy demands. Those effects have probably an even stronger impact in reproductive years, in which dormice were more active. Although these patterns could be found in all areas, there were also considerable differences in average survival rates, with resulting differences in mean lifetime reproductive success between populations. Our results suggest that edible dormice have adapted their life history strategies to maximize lifetime reproductive success depending on the area specific frequency of seeding events of trees producing energy-rich seeds.

The factors explaining changes in population size are a central theme in ecology, and assessing vital rates, i.e. rates of birth, recruitment and mortality in long-term population studies is essential to understand population dynamics and life history trade-offs (Gaillard et al. 1998, Caswell 2001, Engen et al. 2009). As optimal energy allocation to a certain trait (e.g. maintenance or reproduction) is not fixed for all individuals or at all times, vital rates may profoundly vary even within a population. The temporal variation of demographic characteristics is of particular interest to ecologists, as it reflects how biotic (e.g. predation pressure) and abiotic factors (e.g. climate) affect vital rates directly or influence the strength of life history trade-offs. In species inhabiting large areas, we can further expect that differences in environmental conditions cause spatial variation in demographic and vital rates. However, empirical studies investigating the variability in vital rates on a large scale are still not common, especially in small mammals. Here, we use the edible dormouse *Glis glis* as a model species to investigate the causes and consequences of variation in vital rates, particularly in their survivorship.

The edible dormouse is an intriguing organism to study these questions, because despite their relatively small size (∼80–120 g) both females and males do not reproduce every year (Bieber 1998, Schlund et al. 2002, Pilastro et al. 2003). Reproduction in this hibernator is strongly linked to the availability of energy-rich food like beechnuts or acorns in late summer/fall. The production of those seeds occurs at irregular intervals and while dormice have a high reproductive rate during mast-seeding years of beech and oak, whole populations can skip reproduction in years of mast failure (Bieber 1998, Schlund et al. 2002, Pilastro et al. 2003). Further, it has been shown that in non-reproductive years survival rates in this species are twice as high as in reproductive years (Ruf et al. 2006). This indicates a strong trade-off between reproduction and future survival that is detectable even at a population-level. Consequently, the frequency of mast seeding events (and thus reproductive years) affects the lifespan of the animals. While dormice in a German population had a mean life span of 3.4 yr (Ruf et al. 2006), dormice in a study area in northern Italy, where seed-masting years occurred more rarely, lived on average for 9 yr (Pilastro et al. 2003).

The actual causes of impaired survival in the interval following reproductive bouts, i.e. the mechanism underlying the reproduction-survival trade-off in this species, is however unknown. We envisioned two principal reasons for increased mortality associated with reproduction. First, reproduction involves increased foraging activity, which will enhance the risk of exposure to predators (possibly with some differences between sexes). Predation is generally known to influence markedly survival rates of small rodents (Norrdahl and Korpimäki 1995, Ims and Andreassen 2000, Bryant and Page 2005, McCleery et al. 2008). For instance, it has been shown for some other hibernators that high mortality rates occur during the active season due to predation (Neuhaus and Pelletier 2001, Meaney et al. 2003, Bryant and Page 2005). A second, but not mutually exclusive hypothesis is that the depletion of energy stores during energetically costly reproductive years increases mortality rates during the hibernation season. For example, some marmot species have been found to suffer from high mortality rates during the hibernation period (Armitage and Downhower 1974, Arnold 1990).

Therefore, we hypothesized that determining the detailed seasonal pattern of survival rates should provide insights into primary causes of mortality. For example, if survival was lowest during the active season, this would suggest predation as the main mortality cause, whilst low winter survival would indicate insufficient energy reserves as the predominant cause of death. However, it is also likely that mortality is not the result of a single factor alone and that several interacting factors are responsible for an individual's death. Vital rates, such as survival, in any particular population may, however, be affected by specific local conditions, such as population density or local predation pressure (Sinclair and Pech 1996, Rood and Reznick 1997, Ciannelli et al. 2007, Sanz-Aguilar et al. 2009). Further, environmental factors were shown to have a strong impact on vital rates and population dynamics (Neuhaus et al. 1999, Farand et al. 2002, Bowman et al. 2005, Schwartz and Armitage 2005). Especially in species with a large geographic distribution, local populations have to cope with diverse environmental (e.g. climatic) conditions. Therefore it may be misleading to generalize conclusions based on vital rates determined in a single population, and clearly investigations on different local populations seem necessary. Also, despite of being separated by large distances, many populations are known to show synchrony in their abundance or other time-varying characteristics (Bjørnstad et al. 1999, Lundberg et al. 2000, Liebhold et al. 2004). Thus, analysis of multiple populations does not only increase the sample size, it also allows investigating geographical variation as well as the temporal synchrony in survival rates of populations (Grosbois et al. 2008). Further, to analyze data from several populations simultaneously represents an important approach if we, for instance, consider the problem of climatic change (Grosbois et al. 2008). However, due to differences in methods and in the analysis of the data it is often difficult to compare results of independent studies. Despite the necessity to investigate multiple populations of one species in different habitats with consistent methods, this is something hardly ever done previously (but see Ciannelli et al. 2007, Sanz-Aguilar et al. 2009).

We used a total of 40 investigation years of capture–recapture data from five populations of edible dormice collected over a large geographical range of their distribution in Europe (Austria, Czechia, England, Germany and the Italian Alps) to determine both seasonal patterns of local survival and their relation to reproductive activity. Specifically, we examined 1) whether there is a common seasonal pattern in survival rates, 2) if and when there are sex differences in survival, 3) whether populations in different habitats differ in their mean long-term survival rates 4) whether mean survival rates are influenced by climatic factors and 5) whether the strength of reproduction–survival trade-offs (and hence mean lifetime reproductive success) depends on local environmental factors, such as the average frequency of mast seeding of trees. For this purpose, we not only used data from comparable field studies but included all recapture histories from all populations into a single, comprehensive Cormack–Jolly–Seber type model (Lebreton et al. 1992), an approach that, to our knowledge, has not been used before. Our results therefore allow a first insight into species-characteristics of life-history traits versus-population specific variation across a large geographical scale.

## Material and methods

### Study areas and data collection

**Figure 1:**
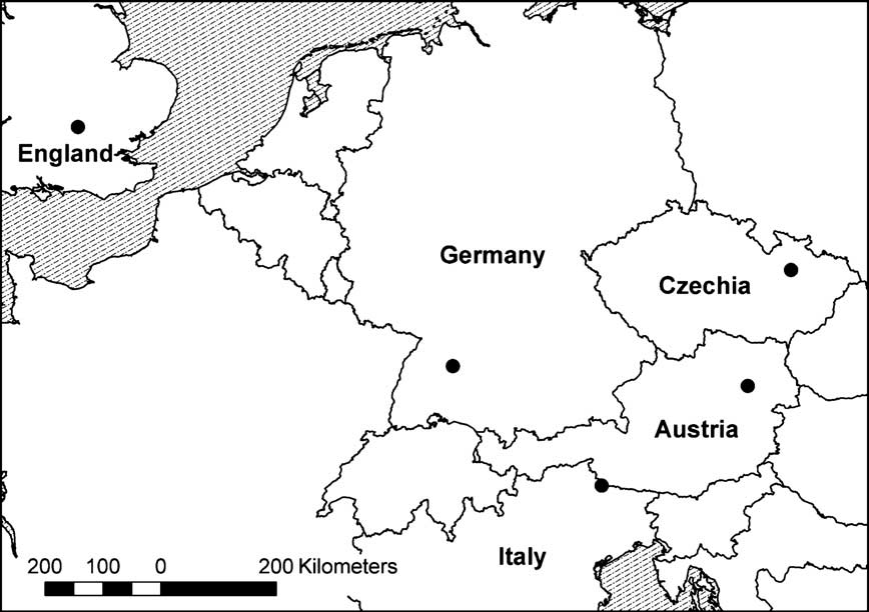
Map of central Europe. The points mark the locations of the studied populations.

We compared local survival probability of edible dormice from study areas in Austria, Czechia, England, Germany and the Italian Alps (Fig. 1, Table 1). Study areas were all dense, seminatural mixed forests, mostly dominated by beech *Fagus sylvatica*, with the exception of Czechia, where oak *Quercus petraea* was the main tree species. In all study areas nest-boxes have been installed, which are used by edible dormice during their active season to rest during daytime and to rear their young. The nest-boxes in Austria and England were put up in lines, at the other areas they were arranged in grid-patterns. Nest-boxes were checked for the presence of edible dormice at regular intervals (depending on the area) during daytime with constant frequency throughout the whole active season (Table 1). Newly captured dormice were sexed and marked individually with subcutaneously injected PIT-tags (Austria, Czechia, England and Germany) or using toe clipping and ear tags (Italian Alps, see Pilastro et al. 2003 for details). As females breed within the nest-boxes offered, the litter size during lactation could easily be assessed and was recorded. Litter size includes juveniles from the day they are born until they are able to leave the nest (i.e. at age 0–40 d). Dormice can be reliably classified as juveniles, yearlings (after their first hibernation) and adult (after their second hibernation) individuals from their size, tibia length and fur color (Schlund 1997, Bieber 1998). However, at the study site in England only a discrimination between juveniles and older individuals was made. We therefore used for our analysis the combined data from yearling and adult dormice and did not test for age effects. We found no evidence for significant differences in population density between the areas (Supplementary material Appendix 1).

**Table 1:** Characterization of the study areas

	Austria	Czechia	England	Germany	Italian Alps
Latitude	48°05′N	49°49′N	51°48′N	48°33′N	46°04′N
Longitude	15°55′E	17°12′E	0°39′W	8°59′E	12°25′E
Altitude (m a.s.l.)	650	380	200	475	1050
Study area size (ha)	1860	23	50	12	60
Fraction of study site border representing a barrier (%)	0	30	20	20	0
Distance to next urban area (>1000 inhabitants) (km)	5.5	1.3	1.0	0.7	6.4
Main tree species	*Fagus sylvatica* (65%)	*Quercus petraea* (45%)	*Fagus sylvatica* (60%)	*Fagus sylvatica* (58%)	*Fagus sylvatica* (80%)
	*Picea abies* (14%)	*Fagus sylvatica* (30%)	*Picea abies* (30%)	*Pinus sylvestris* (16%)	*Picea excelsa* (10%)
Mean tree age (yr)	40–95	20–110	20–70	–	–
Sum of marked individuals	1070	304	473	619	1356
Number of nest-boxes	197	100	135	126	100
Interval between nest-box controls	2 weeks	1 week	4.5 weeks	1 week	2 weeks
Mean duration of each capture occasion (days)	3	1	1	1	2
Study period	2006–2008	2006–2008	1996–2008	1993–2005	1991–1998
Years without (or very low) reproduction	–	–	1996, 1998, 2003, 2005, 2008	1993, 1996, 1997, 2000, 2002, 2004, 2005	1993, 1994, 1996, 1997, 1998

### Survival estimation and statistical analysis

The active period lasted from May to September in Austria, in the other areas from June to October with a few outliers before and after those five months. To obtain uniform capture histories we pooled the data from the original capture events to five monthly capture occasions, assigning captures from outliers to the first or fifth month, respectively. Those were used to model survival (F, the combined effect of mortality and emigration) and recapture probabilities (p) with Cormack–Jolly–Seber models (Lebreton et al. 1992). We used the R (R Development Core Team 2009) package RMark (Laake and Rexstad 2008) to construct models for program MARK 5.1 (White and Burnham 1999). In our analysis we estimated the effects of local differences (‘Area’), sex, month in the active season (‘Month’) and reproduction (‘Repr’) on survival. For the factor reproduction we differentiated between years with above- and below-average reproduction in each area as determined by the number of juveniles captured per year. This classification of the reproductive years was largely based on (individually marked) juveniles at the stage of weaning, and older juveniles (independent from their mother) made up only a much smaller fraction. As the total number of juveniles captured and the number of reproductive females is highly correlated (German population: r=0.929, p<0.001, DF=11), we are confident to indeed have measured reproduction and not reproductive success (i.e. recruitment of juveniles) with this classification. We hereafter label years as reproductive years (RY) and non-reproductive years (NRY). In Austria and Czechia we had a relatively consistent high proportion of reproducing individuals and high numbers of juveniles throughout the whole study period. We therefore classified all years in those areas as RY. The results for differences between RY and NRY are therefore based only on the data from England, Germany and Italy. The pattern of reproductive years varied between the study areas (Table 1). In a further step we presumed that monthly survival (the survival from one month to the next) was constant during early summer (ES) and during late summer (LS). We called this factor ‘Season’, with levels ES, LS and W (winter: survival probability from the last month in late summer to the first month in early summer of the following year). As the last estimate for survival and recapture probability cannot be separated, we created a distinct factor-level for the very last occasions for the factors ‘Month’ and ‘Season’, which is not included in the results shown. We tested for additive effects (‘+’) as well as for interactions (‘×’). To test for the goodness of fit of our general model [F(Area×Sex×Month×Repr) p(Area×Sex×Month×Repr)] we used the parametric bootstrap approach implemented in program MARK. Based on 1000 bootstrap replicates from a random sample of 500 (out of 3695) capture histories, this test suggested that our general model fits the data (p=0.608). This restriction to 500 capture histories was necessary because the computations on the full data set exceed the available computer capacity. In addition, we tested for overdispersion of the global models using the median-ĉ approach also implemented in program MARK.

Emanating from the general model we created more parsimonious models where the factor ‘Season’ instead of ‘Month’ was used and/or models with fewer factors. First, we fixed the survival estimates to the fully parameterized model [F(Area×Sex×Month×Repr)] and created all possible models for recapture probability. From those we chose the model which represented the data best with a minimum number of parameters. In a second step we used the configuration for p from this model and constructed models with all possible factor combinations for F. For model selection we used the quasi-likelihood corrected (ĉ=1.157) Akaike information criterion (QAIC, Akaike 1973, Burnham and Anderson 2002). All models within a difference of a ▵QAIC<2 from the best model were considered, as they have a substantial level of empirical support (Burnham and Anderson 2002).

In addition to the general model including area as a factor, we also calculated separate models for each area to compare if those factors that remain in our best model, and hence seemed important, can also be found in the best models computed independently for each area (Supplementary material Appendix 2). These individual area models were also used to investigate possible trends in temporal variation of survival (for data sets>3 yr).

**Figure 2:**
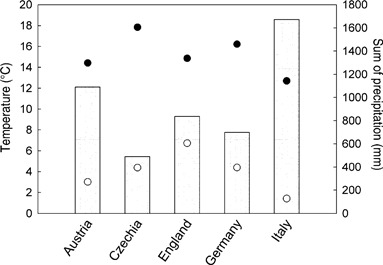
Mean ambient temperature during the active season from May to September (black dots) and the hibernation period from October to April (white dots). Gray bars represent the yearly sum of precipitation.

By replacing the factor ‘Area’ in the general model with the lowest QAIC with possible influential environmental variables we examined which area-specific factors may affect survival rates. We tested for the possible effects of climatic variation by averaging monthly mean temperature during the active season (May–September, ‘TempA’), the average monthly mean temperature during the hibernation season (October–April, ‘TempH’) and the yearly sum of precipitation (‘Prec’) over the study period (Fig. 2). We included ambient temperature as a possible important environmental signal, because in edible dormice ambient temperature strongly affects annual biological cycles, particularly gonadal development (Jallageas et al. 1989). Precipitation influences thermal conductance and could therefore cause an increased heat loss in the animals; therefore we included this factor as well. Since ambient temperature determines energy expenditure during hibernation, a low temperature during this period could impair survival (Heldmaier et al. 2004), which led us to include TempH. Further, low ambient temperatures and rain may also influence food availability. Weather data were provided by the Lower Austria state government (department for hydrology) for the study site in Austria, from the Czech Hydrometeorological Inst. for the study site in Czechia, from Meteorological Office for the study site in England, from the Meteorological station Herrenberg (means from 1996–2005 only) for the study site in Germany, and from the Meteorological Service of the Regione Veneto for the study site in the Italy. Please note that although the study site in Italy was the southernmost, it is located in the Alps (1050m a.s.l.) and was by far the coldest and rainiest of all study areas (Fig. 2). Again, we used the QAIC to identify the model with the highest explanatory value.

Mean monthly survival rates (for each sex and differentiated between RY and NRY) during the active period (ES and LS) over all areas were computed and their confidence intervals were calculated using the delta method (Oehlert 2010). The yearly survival rates in RY (F_RY_) and in NRY (F_NRY_) were computed by multiplying all monthly survival rates, again we used the delta method to calculate confidence intervals. Following Seber's formula for the mean life expectancy (E_L_=−1/log(F), Seber 1982), yearly survival rates were used to estimate mean life span after the animal's first hibernation (MLS_1_) depending on sex, area and the proportion of reproductive years (r_RY_):


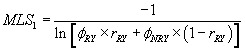


This estimation includes the assumption that for each area the survival rates are stable at different proportions of reproductive years. The variance of the MLS_1_ was also estimated according to Seber (1982), and we further calculated standard deviation as the square root of the variance. To obtain the total mean life span (MLS) we added one year (survival as juvenile) to MLS_1_. This estimate of MLS as a function of reproduction frequency could not be computed for the populations in Austria and Czechia because in those areas dormice reproduced in all years. We calculated the mean litter size per female (ls) for each area and used ANOVA to compare litter sizes between the areas. Further, we estimated the expected mean lifetime reproductive success (LRS) of the females in England, Germany and Italy:


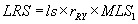


For the analysis of the MLS, LRS and litter sizes we used the statistical software R (R Development Core Team 2009).

## Results

### Model selection

Modeling the recapture probability estimates when the survival estimates were fixed to the full model showed that the fully parameterized model p(Area×Repro×Month×Sex) was by far the model that fitted best. The difference in the QAIC to the second best model with p(Area×Repro×Month) was 105.5. Therefore, recapture probability was fixed to the full model when survival was estimated. Survival rates were best described by F(Area×Repro+Season+Sex). All other models had a ▵QAIC>2 compared to this model and were therefore not considered (Table 2a). Consequently, for our further analysis we used only estimates from the most likely model: F(Area×Repr+Season+Sex) p(Area×Sex×Month×Repr).

**Table 2:** (a) Model selection for survival estimates and (b) model selection for survival estimates considering possibly influential environmental factors (‘TempA’– mean temperature during active season, ‘TempH’– mean temperature during hibernation, ‘Prec’– yearly sum of precipitation). Estimation of the recapture parameters was fixed to the model p(Area×Repro×Month×Sex). Models are ranked according to their QAIC. np number of estimated parameters, QAIC quasi-likelihood corrected AIC, ▵QAIC difference between the QAIC and the minimum QAIC, Model likelihood relative strength of evidence for a model within the set of models computed. Note that not all calculated models are shown

(a) Survival estimates						

Rank	Survival parameters φ	np	QAIC	▵QAIC	Model likelihood	Deviance
1	Area×Repro+Season+Sex	161	22699.50	0.00	0.83	11778.23
2	Area×Repro+Month+Sex	163	22702.73	3.24	0.17	11777.47
3	Area×Repro×Sex×Season	173	22714.24	14.74	<0.001	11768.97
4	Area×Repro+Month	175	22717.48	17.99	<0.001	11768.22
5	Area×Repro+Season	162	22717.73	18.23	<0.001	11794.47
8	Area×Repro×Season	190	22736.15	36.66	<0.001	11756.89
9	Area+Repro+Season+Sex	155	22748.62	49.12	<0.001	11839.35
28	Area×Repro×Season×Sex	236	22782.82	83.33	<0.001	11711.56
48	Area×Repro×Month×Sex	288	22871.08	171.58	<0.001	11695.82
79	constant	145	23557.72	858.23	<0.001	12668.46

(b) Environmental factors						

Rank	Survival parameters φ	np	QAIC	▵QAIC	Model likelihood	Deviance
1	Area×Repro+Season+Sex	161	22705.38	0.00	0.99	11778.23
2	TempA×Repro+Season+Sex	154	22714.91	9.53	0.01	22401.52
3	Prec×Repro+Season+Sex	154	22727.06	21.68	<0.001	22413.67
4	TempH×Repro+Season+Sex	154	22955.05	249.67	<0.001	22641.66

### Survival probability

**Figure 3:**
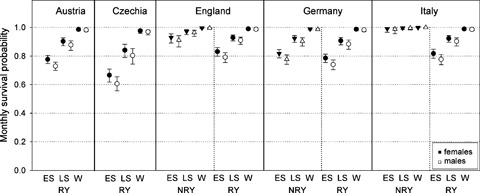
Local monthly survival probability ±95% CI at the five study areas depending on the season (ES – early summer, LS – late summer, W – winter), sex and differentiated by reproductive (RY) and non-reproductive years (NRY). Note that in Austria and Czechia only reproductive years occurred during the study period.

**Figure 4:**
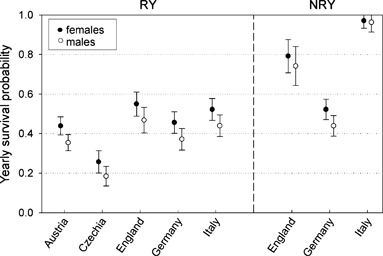
Yearly survival probability ±95% CI at the five study areas, differentiated by reproductive (RY) and non-reproductive years (NRY). Note that in Austria and Czechia only reproductive years occurred during the study period.

There was a clear seasonal pattern in the survival rates of edible dormice. Monthly survival was lowest during ES, higher during LS and highest during winter (Fig. 3). This pattern was found in all areas, both sexes and in RY as well as in NRY. Non-overlapping 95% confidence intervals reveal that this difference was significant (p<0.05) for all areas during RY, in the study site in Germany also for NRY. In NRY the seasonal pattern was less distinct, especially at the study site in the Italian Alps. Mean survival rate differed between the study sites, and was lowest at the study site in Czechia, especially in ES. As expected, monthly survival was higher in NRY, but the impact of this factor depended on the study area (Fig. 3, 4). The difference between RY and NRY was most distinct and significant (p<0.05) for all seasons at the study site in Italy, while the difference in Germany was less pronounced. Comparing the resulting yearly survival rates revealed significantly (p<0.05) different survival rates between RY and NRY in England and Italy (Fig. 4). Yearly survival during the RY was significantly lower (p<0.05) at the study site in Czechia compared to all the other areas, which had rather similar survival rates. However, the yearly survival rates in the NRY differed significantly (p<0.05) between areas (Fig. 4).

At all study sites and in RY as well as in NRY, female dormice always had slightly higher survival rates than males (Fig. 3, 4). The effect size of the difference between the sexes was rather small, and there was no clear significant difference between males and females. However, if the estimates over all areas were averaged, females had during the active period a mean monthly survival rate of 0.84 (CI: 0.82–0.85) in RY and 0.94 (CI: 0.93–0.95) in NRY, whereas the mean monthly survival rates in males were 0.80 (CI: 0.78–0.82) in RY and 0.92 (CI: 0.91–0.94) in NRY.

The models estimated separately for each area showed the same general pattern as the resulting model for all areas (Supplementary material Appendix 2). In particular, there was a strong seasonal pattern in all areas with low survival in early summer, higher rates in late summer and the highest survival rates in winter. For all of the populations experiencing RY as well as NRY, the factor reproduction was retained in the best models (models with a ▵QAIC<2 from the model ranked first), and again, survival was higher in NRY compared to RY. The factor sex was included in the best models in all areas, except for the population in Czechia. Further, we found no evidence for any long-term trend in the survival rates over the years.

### Recapture probability

**Figure 5:**
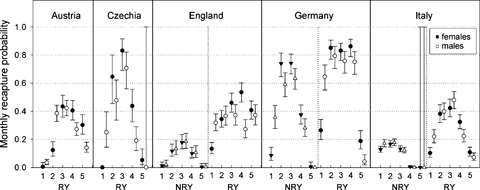
Local monthly recapture probability ±95% CI at the five study areas depending on the month in the active season (1–5), sex and differentiated by reproductive (RY) and non-reproductive years (NRY). Note that during the study period in Austria and Czechia only reproductive years occurred.

The recapture probability in the studied dormouse populations differed markedly, depending on area, sex, month in the active season, and between RY and NRY (Fig. 5). The general pattern was a very low recapture probability at the beginning and at the end of the active season, and a higher recapture probability in the middle of the active season. Within each area the recapture probability was higher in years with high reproduction.

### Climatic factors

None of the investigated climatic factors could explain the spatial variation in the survival rates better than the factor ‘Area’ (Table 2b). All models including weather data had a ▵QAIC>2 and could therefore be disregarded.

### Average lifespan and lifetime reproductive success

Comparing the MLS regarding the actual number of reproductive years within an area, edible dormice had a MLS±SD of 3.3±1.3 yr for females and 2.8±1.0 yr for males in England (61.6% RY), 2.4±0.8 yr for females and 2.1±0.6 yr for males in Germany (53.8% RY), and 5.6±2.5 yr for females and 4.8±2.1 yr for males in Italy (37.5% RY).

There was a significant difference in the number of juveniles per litter between the areas (F_4,521_=9.840, p<0.001). However, the effect size was rather small, as mean litter size±SD was 5.4±1.7 (n=77) in Austria, 6.1±1.2 (n=55) in Czechia, 5.4±2.37 (n=162) in England, 6.3±1.6 (n=150) in Germany and 5.0±1.6 (n=82) in Italy.

**Figure 6:**
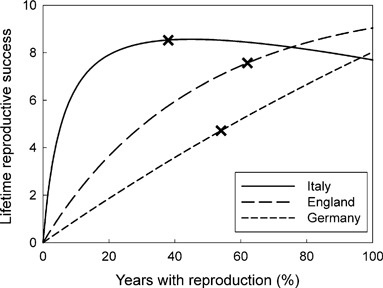
Estimated lifetime reproductive success (number of juveniles) as a function of the proportion of years with reproduction (under the assumption that the survival rates are stable at different proportions of reproductive years). The crosses mark the actual proportion of reproductive years found in each area during the study period.

In England and Germany the estimated LRS increased with the proportion of RY, with the values for England remaining always greater than those for Germany (Fig. 6). For Italy there was a much steeper course, resulting in a high LRS even at a low proportion of reproductive years. However, in contrast to the other two areas, there was a small decrease in LRS as the proportion of reproductive years increased (Fig. 6). For the actual proportion of reproductive years occurring during the study period we estimated an LRS of 7.6 juveniles in England, 4.7 juveniles in Germany and 8.5 juveniles per lifetime in the Italian Alps.

## Discussion

### Seasonal survival

In small rodents causes of mortality are often difficult to assess, but determining the most precarious times and differences in survival rates within and between populations can help to identify mortality causes. The very high survival rates during winter in all populations indicate that adult edible dormice typically did not die due to insufficient energy reserves during hibernation. Although hibernation is often considered the most crucial time, a high winter mortality has in fact only been shown for a few hibernators (Armitage and Downhower 1974, Arnold 1990), whilst most studies found high survival rates during hibernation (Neuhaus and Pelletier 2001, Schaub and Vaterlaus-Schlegel 2001, Meaney et al. 2003, Bryant and Page 2005, this study). An obvious reason for high winter survival rates is that animals in their hibernacula are relatively secure from predators.

Adult edible dormice lose about 30% of their pre-hibernation body mass during hibernation (Fietz et al. 2005), and after emerging from hibernation they can suffer from depleted energy reserves. They therefore need to spend an increased time on foraging, which arguably makes them more perceivable to predators and thus increases their mortality rate. Particularly adverse is the fact that most of the species predating on edible dormice rear their young early in summer (e.g. tawny owl *Strix aluco*, Eurasian eagle-owl *Bubo bubo*), and have increased energetic demands during this time period (Bauer et al. 2005).

Using Cormack–Jolly–Seber models it is theoretically not possible to discriminate between mortality and emigration, which could also explain seasonal differences in the survival parameters. However, while juveniles may emigrate, yearling and adult dormice show an extremely high site fidelity (Vietinghoff-Riesch 1960, Ruf et al. 2006, Bieber and Ruf 2009), and it is therefore very unlikely that our survival estimates were confounded by emigration.

### Sex-specific survival

As the highest reproductive costs for males arise during the mating period in early summer (Bieber 1998, Fietz et al. 2004, Lebl et al. 2010) and for females during lactation in late summer (Kager 2004, Zoufal 2005), we had predicted different seasonal pattern of survival in the two sexes. Contradicting our expectations, there was a constantly lower survival in males, which was consistent for all seasons and areas. However, this difference in the survival rates between females and males was relatively small. The fact that this difference in survival rates persisted through a longer time period (and not only during the mating period) suggests that it may be due to the negative effects of androgens on male immunocompetence (Kraus et al. 2008). However, presently we cannot rule out that alternatively, males may be more active, with associated increased predation risk, throughout the whole summer. The larger home range size in males indicates a higher activity level (Jurczyszyn and Zgrabczynska 2007, Scinski and Borowski 2008). Further, body mass of males remained lower in RY than in NRY throughout July and August in the Italian population (Pilastro et al. 2003).

### Implications of reproduction

As expected, the mortality rates in the energetically challenging RY were higher, as already shown for the population in Germany (Ruf et al. 2006). Females need to assimilate during lactation up to 50% more energy than non-reproducing females (Zoufal 2005) and sexually active males have been found to lose up to 30% of their body mass during the mating phase (Bieber 1998, Fietz et al. 2004, Lebl et al. 2010). As outlined by Bieber (1998), intramale competition, which involves considerable energy expenditure for locomotion, and/or low food intake, could explain this body mass decline. Sexually quiescent males also increase their time spent in daily torpor and at least part of the body mass loss in reproductively active males could be attributed to higher thermoregulatory costs (Fietz et al. 2004). Another benefit of increased daily torpor could be a reduced predation risk since animals using daily torpor need less energy and thus less time spent on nocturnal foraging. Torpor as a mechanism for predator avoidance is also likely for northern long-eared bats *Nyctophilus bifax*, which readily use torpor even during periods of high food availability and good body condition (Stawski and Geiser 2010). Edible dormice have also been found to exploit this benefit and use prolonged periods of summer dormancy (i.e. estivation in underground dwellings as during hibernation) and can spend altogether more than 10 months yr^−1^ in dormancy (Bieber and Ruf 2009). Summer dormancy could explain higher survival as well as lower recapture rates during NRY. Consequently, the less pronounced difference in the survival and recapture rates of the German population between RY and NRY in comparison to England and Italy may indicate a lower use of summer dormancy.

Dormice not only base their yearly reproductive decision on the occurrence of energy rich seeds (e.g. beech, oak), our results show that they are also able to adapt their life history strategies to maximize their LRS depending on the frequency of mast seeding events in a specific area. Those adaptations are reflected by the differences in the survival rates, but also by slight differences in litter size. Compared to Italy and England, dormice in the German population had rather low survival rates. However, although we found the highest mean litter size in this area, which may be an adaptation to counteract the lower survival rates, animals in the German population were not able to reach an LRS similar to that in the Italian population. Good years for reproduction were rare in the Italian Alps, but the population inhabiting this area reached their maximum LRS exactly at the observed frequency of mast years. It is therefore possible that the dormice of the population in the Italian Alps are particularly adapted to use predator avoidance strategies and/or allocate increased amounts of energy to somatic maintenance and repair in non reproductive years compared with other areas (Kirkwood 1977). In England and Germany mast years of beech occurred at much shorter intervals and dormice in those areas reach their LRS at a much higher proportion of reproductive years. The question remains if these adaptations are due to genetic differences between the populations or if phenotypic plasticity allows edible dormice to adjust to the momentary frequency of good masting years.

### Spatial differences

There was no indication that the distribution of the nest-boxes (lines vs grids) influenced the capture probability of dormice, as the recapture probability in Austria and England did not differ systematically from the other areas (Fig. 5). However, the high recapture rates for dormice in Czechia and Germany are likely to be the result of the higher frequency of nest-box controls in these areas. Further, the nest-box density may have also influenced the recapture rates. Although differences in the study designs are likely to bias the area differences in the recapture rates, it has been shown by computer simulations that a possible bias in survival rates caused by heterogeneous capture probabilities is negligible (reviewed in Pollock et al. 1990). Therefore, even if the capture probability was influenced by different designs, survival estimates are still robust.

Although the pattern of seasonal survival was very similar in all study areas, we found a clear distinction in the general level. Associated differences in LRS of ∼45% between Germany and the Italian Alps confirm that environmental conditions can have an important influence on vital rates.

Our results indicate that climatic factors do not explain these differences in mean survival rates between the study areas. This result concurs with many other studies which could not find a connection between climatic factors and survival rates (Farand et al. 2002, Sendor and Simon 2003, Borrego et al. 2008). Further, there was no evidence for a long-term trend in the survival rates over the years, which could have indicated an influence of climatic change on dormouse survival (Supplementary material Appendix 2). Still, we cannot totally exclude the effect of climate since it was confounded with the factor ‘Area’. In addition, climate might have detectable effects on other traits at a much finer scale. For example, at the study site in Czechia, spring temperatures had an influence on the timing of vernal emergence from the hibernacula (Adamík and Král 2008). Thus, climate might still influence survival indirectly, even in unexpected ways. For instance, the low temperatures at the study site in the Italian Alps may increase the frequency of daily torpor and thus lead to positive effects on survival. However, the results of our analysis show that some unidentified factor(s) associated with area difference had a much stronger influence on survival than temperature or precipitation alone.

As yearling dormice invest less in reproduction (Pilastro et al. 1996, Lebl et al. 2010) and may have differing survival rates than adults (Ruf et al. 2006), demographic differences are likely to contribute to the differences in mean survival between the areas. Ruf et al. (2006) found for the population in Germany that including yearlings (many of which skip reproduction in early life) weakens the effect of higher mortality in RY. The extreme difference in the Italian Alps between RY and NRY may therefore partly be due to a lower proportion of yearlings since in this area dormice seldom reproduce in successive years. Unfortunately, the age of the individuals was not assessed in all areas and the local demographic compositions are therefore unknown. In England, reproductive years occur approximately as often as in Germany and thus the proportion of yearlings should be similar in those areas. Hence, the higher survival rates in England compared to Germany may indicate a somehow ‘better’ habitat. This could result in a higher proportion of reproductive yearlings in England and a clearer difference between RY and NRY. Differences in the forest composition and the local structure might also influence survival rates of dormice. In Czechia we found the lowest mean survival rates and only this study site was dominated by oaks instead of beech trees as in the other areas. This might not only be caused by a difference in food (and therefore energy) supply, but may also result in differences in arboreal structures providing cover from predators.

With our present dataset we cannot determine the actual causes of death. However, since predation clearly is the major factor determining life span in rodents of this size (Norrdahl and Korpimäki 1995, Ims and Andreassen 2000, Bryant and Page 2005, McCleery et al. 2008), it is therefore the most likely cause for the different mortality rates between the study areas. For edible dormice the main predators are the tawny owl, Eurasian eagle-owl, pine marten *Martes martes*, least weasel *Mustela nivalis* and cats *Felis sylvestris*, *F. catus* (Vietinghoff-Riesch 1960). All of these predators are likely to occur in all of the study areas, with the exception of England, where Eurasian eagle-owl and pine marten are missing (Mitchell-Jones et al. 1999, Bauer et al. 2005). However, large-scale distribution maps cannot actually quantify local predation pressure which is influenced by small-scale differences in the occurrence and density of certain predators as well as the availability of alternative prey species. Thus, as a working hypothesis for future studies we would consider local predation pressure as a major determinant of survival and population dynamics in edible dormice.

Supplementary material (Appendix E6691 at <http://www.oikos.ekol.lu.se/appendix>). Appendix 1–2.
